# FEASIBILITY AND ACCEPTABILITY OF DETECTING COGNITIVE IMPAIRMENT AND DEMENTIA IN PRIMARY CARE PRACTICES

**DOI:** 10.1093/geroni/igz038.2877

**Published:** 2019-11-08

**Authors:** Rachel O’Conor, Julia Yoshino Benavente, Laura Curtis, Marina Arvanitis, Richard Gershon, Michael Wolf

**Affiliations:** Northwestern University, Chicago, Illinois, United States

## Abstract

As the age of the US population increases, so does cognitive impairment (CI); therefore early detection of CI is critical for ensuring its appropriate management. As part of a NINDS Consortium to detect CI and dementia in primary care (DetectCID), we are implementing and evaluating a brief 2-step CI detection paradigm (MyCog), that can be delivered in clinics with diverse populations via the electronic health record (step 1) and iPad (step 2). We conducted focus groups with 25 clinicians and administrative leaders from academic and community primary care practices to 1) understand how CI is being assessed, and 2) evaluate the feasibility of implementing the MyCog paradigm into existing primary care workflows. Several key themes emerged from the discussions. No proactive detection strategy for CI was regularly used outside of the Medicare Annual Wellness Visits (AWV); variable assessments including the Minicog, MoCA, or MMSE were used to fulfill the AWV requirement. Regarding the feasibility of our MyCog Paradigm, our 2-step process was positively received, with the brief case-finding step 1 satisfying AWV requirements and replacing the longer assessments currently being used. Clinicians preferred that step 2 be self-administered due to limited clinician time for wellness visits, and highlighted logistical challenges such as room availability and storage and maintenance of the iPad. Overall, clinicians felt that the identification of CI was valuable and supported standardization, but indicated regular case finding was unlikely without clear guidance on clinical decision-making.

